# Functional Multipotency of Stem Cells: What Do We Need from Them in the Heart?

**DOI:** 10.1155/2012/817364

**Published:** 2012-08-26

**Authors:** Pablo Díez Villanueva, Ricardo Sanz-Ruiz, Alberto Núñez García, María Eugenia Fernández Santos, Pedro L. Sánchez, Francisco Fernández-Avilés

**Affiliations:** Cardiology Department, Hospital General Universitario Gregorio Marañón, Madrid, Spain

## Abstract

After more than ten years of human research in the field of cardiac regenerative medicine, application of stem cells in different phases of ischemic heart disease has come to age. Randomized clinical trials have demonstrated that stem cell therapy can improve cardiac recovery after the acute phase of myocardial ischemia and in patients with chronic ischemic heart disease, and several efficacy phase III trials with clinical endpoints are on their way. Nevertheless, a complete knowledge on the mechanisms of action of stem cells still remains elusive. Of the three main mechanisms by which stem cells could exert their benefit, paracrine signaling from the administered cells and stimulation of endogenous repair are nowadays the most plausible ones. However, in this review we will define and discuss the concept of stem cell potency and differentiation, will examine the evidence available, and will depict future directions of research.

## 1. Introduction

Cardiovascular disease, mainly represented by coronary heart disease and heart failure, continues to be one of the most significant burdens to healthcare systems in the United States and Europe [1]. In the United States alone, about 1 million patients suffer an acute myocardial infarction (AMI) every year, with more than 7 million survivors at present, many of them susceptible of developing heart failure. Thus, up to 5 million people are living under congestive heart failure (CHF) conditions, with an inherent associated 20% mortality per year. Recent advances in medical and device therapy for ischemic heart disease and heart failure have greatly improved both prognosis and quality of life of these patients. Pharmacological and no pharmacological strategies that enhance myocardial perfusion, increase electrical stability of the myocardium, limit ventricular remodeling, and improve ventricular function have been developed, but hospitalization and mortality rates are still high and lead to an overwhelming cost. Cardiac transplantation is sometimes the only therapeutic approach in patients with end-stage CHF, but demand exceeds the availability and suitability of donor hearts. This was the starting point to the search for new therapeutic modalities.

Thus, over the last two decades, several animal studies and some clinical trials supported the use of cardiac cell therapy as a potential and alternative therapeutic modality, offering the possibility of repairing the damaged tissue and restoring its normal function, either after an AMI or in the chronic phase of coronary artery disease (CAD) [2]. In the beginning, this therapy was conceived to repopulate the damaged myocardium by the transformation “differentiation” of donor stem cells into new functional cells. This potential was demonstrated in several experimental studies and preclinical models, in which embryonic and adult stem cells were shown to differentiate into cardiomyocytes, vascular smooth muscle cells, and endothelial cells. The so-called “regenerative therapy” for cardiovascular disease had born [3].

However, the excellent results obtained in the preclinical models never translated with the same efficiency in the bedside [4, 5]. Although phase I and II randomized clinical trials (RCTs) indicate that cell therapy is a safe treatment which can improve cardiac function after an AMI and in the chronic phase of CAD; trial results are not uniform due to (1) a lack of standardization of cell isolation and delivery procedures, (2) the absence of a universally accepted nomenclature, and (3) the large number of stem cell types under investigation in different clinical settings [4]. Moreover, a deeper knowledge on the mechanisms of action of stem cells in humans is warranted to clarify the controversial results obtained after RCTs [6]. One of the mechanisms of action, differentiation of stem cells into mature functional adult cells, will be the aim of this paper.

## 2. The Concept of Functional Potency as a ****Mechanism of Action of Stem Cells

Until recently, most attention in cardiac research has focused on the pathophysiology of coronary atherosclerosis and left ventricular remodeling and dysfunction after a myocardial insult, with little attention paid to the concept of cardiac repair. This was so because of the widely accepted concept that the heart was a terminally differentiated organ, notion that changed after the discovery of certain immature progenitor cells involved in the process of cardiac repair: bone marrow-derived cells, circulating endothelial progenitor cells (EPCs), and finally resident cardiac stem cells [2].

“Stem cells” are undifferentiated self-replicating cells capable of generating, sustaining, and replacing terminally differentiated cells [7]. In other words, they remain immature in an early stage of development, capable of dividing ad infinitum (self-renewable) leading to both identical cells and different cell lineages (potency), with capability for functional regeneration of tissues. Broadly speaking, stem cells can be divided into two principal groups, embryonic stem cells (totipotent or pluripotent) and adult stem cells (multipotent). Another cell type, “embryonic-like” cells generated after genetic manipulation of mature cells (induced pluripotent cells, iPS) will also be commented in this paper.

### 2.1. Totipotency, Pluripotency, and Multipotency

Fertilization triggers the first cell division of the embryo [8, 9]. Until the 8-cell stage, all cells in the embryo are totipotent. We can define a totipotent cell as one that can give rise to a new individual if provided with appropriate maternal support. The small cluster of cells inside the blastocyst, the inner cell mass (ICM), is destined to give rise to all the tissues of the body. From that stage, embryonic genome undergoes activation and the cells of the ICM are pluripotent but not totipotent [10].

Pluripotent cells can give rise to all tissues of the body plus many of the cells that support the pregnancy but are unable to produce a new individual on their own [11]. They are diploid and karyotypically normal cells which can be propagated indefinitely in the primitive embryonic state. These cells can differentiate spontaneously at high frequency under a range of conditions into multiple-cell types representative of all 3 embryonic germ layers, *in vitro* and *in vivo* [12]. An important additional criterion for pluripotentiality is the demonstration that the stem cell line may be cloned from a single cell [10].

**Figure 1:**
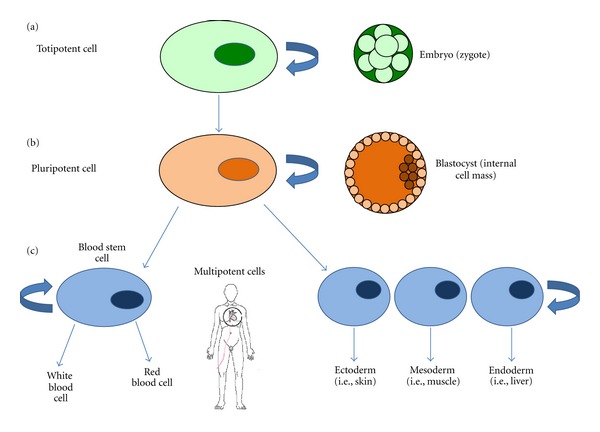
Schematic representation of the functional potency of stem cells. (a) Totipotent cells are present in the embryo until the 8-cell stage. They can generate all cells of the organism and even a whole new individual if provided with appropriate maternal support. (b) Pluripotent cells reside in the internal cell mass of the blastocyst. During the life of the embryo and as tissues differentiate, pluripotency extinguishes. They can differentiate into multiple-cell types representative of all 3 embryonic germ layers. (c) Once the organism is formed, multipotent cells are present in all tissues, committed to ectodermic, mesodermic, or endodermic differentiations. The turning arrow indicates the clonogenic self-renewal capacity of stem cells.

Once stem cells are confined in a certain tissue, they become multipotent “adult stem cells” [7]. In other words, they undergo asymmetric self-renewing cell divisions but have less self-renewal ability, in part because of lack of high levels of telomerase. In addition, the array of differentiated cells that can be generated from adult stem cells is more limited, and these cells generate daughter cells that can differentiate into cells of the tissue of origin but not into another cell types [13, 14] (Figure 1).

### 2.2. Plasticity or Transdifferentiation

Over the past 8 years, a series of reports have been published suggesting that the previous dogma of tissue specificity associated with adult stem cells may not be correct [15]. Elegant studies of chimerism opened the door to this revolutionary concept in cardiovascular medicine [16]. In these studies, the existence of an extracardiac stem cell population (probably original from the bone marrow) with capacity to contribute to damage reconstitution after an injury in transplanted hearts was suggested. Male patients, which received a heart from a female donor, showed Y chromosome-positive cells both in the myocardium (cardiomyocytes) and in the vessel wall (smooth muscle and endothelium). Those findings of primitive cells from the recipient (XY) in the grafted heart (XX) demonstrated that progenitor cells from an extracardiac origin migrated and engrafted into the heart early after transplantation, undergoing differentiation and acquiring functional competence thereafter [17].

This presumed ability of tissue-specific stem cells to acquire the fate of cell types different from the tissue of origin has been termed plasticity or transdifferentiation [12, 18–20]. Through this process, tissue-specific multipotent adult stem cells thought to be committed to a given cell lineage can under certain microenvironmental conditions acquire the ability to differentiate into cells of a different tissue [21]. Different conditions (i.e., ischemic tissue injury) may act as a trigger for these cells, promoting the appearance of certain cellular mediators that work as “reprogramming factors” and leading to a change in gene expression in the cell nucleus (processes of de- and redifferentiations). However, the ability of these new transdifferentiated cells for self-renewal has not been unanimously proved experimentally. Furthermore, other mechanisms that may explain plasticity, like fusion of the donor cells with resident cells in an organ or the existence of true pluripotent stem cells in postnatal life, have not been demonstrated consistently in humans [22, 23] (Figure 1).

### 2.3. Other Mechanisms of Action

Although it is beyond the scope of this paper, it is important to mention that other mechanistic explanations of stem cell therapy benefit are nowadays more plausible and more accepted than transdifferentiation: firstly, the release of factors is capable of paracrine signaling from the administered cells: secondly, the stimulation of endogenous repair by injected cells through cardiac stem cell niches activation [24].

## 3. Potency of Different Types of Stem Cells

### 3.1. Location of Stem Cells: Activation of the Intrinsic Mechanisms of Repair

There is significant evidence that adult multipotent stem cells are present in several adult tissues, not only confined to tissues with rapid cell turnover, such as skin and epithelial mucosa or hematopoietic tissue [25]. Accordingly, recent studies reveal the presence of highly plastic stem cells in bone marrow that can lead to cells from all three embryonic germ layers. Bone marrow stem cells, especially hematopoietic stem cells (HSCs) and mesenchymal stem cells (MSCs), are known to posses the ability to differentiate into different cell lineages, such as liver, skin, intestine, skeletal muscle, cardiac muscle, and even neural derivates [9]. Other cells like bone-marrow-derived macrophages can also transdifferentiate into a cardiomyocytic phenotype under certain conditions when cultured in myocardial tissue.

Immune response activation also seems to be involved in cell regeneration [26]. The inflammatory cascade that characterizes ischemic myocardial injury has been related to the differentiation of monocytes into endothelial cells and to the mobilization of other cell populations to the damaged tissue [27, 28].

On the other hand, resident cardiac stem cells have also been implicated in the process of cardiac repair. The myocardium is supposed to be able to repair itself, due to convincing data supporting the existence of these cells, and able to regenerate myocytes and vasculature after damage. However, this capacity for self-repair is limited in hearts that have suffered an acute injury or that are chronically failing. The heart's endogenous regenerative capacity seems to be not enough to face and repair the massive loss of cardiomyocytes after being hurt [25]. Thus, transplantation of exogenous adult stem cells in order to enhance and potentiate this self-repair process is the basis for cardiac stem cell therapy.

### 3.2. Embryonic Stem Cells

Embryonic stem cells (ESCs) can be classified into totipotential and pluripotential. The former are present in the earliest stage of embryonic development, in the fertilized oocyte (fertilized egg or zygote), or in the ICM of the blastocyst before the 8-cell stage [9]. They are capable of generating any terminally differentiated cell from any of the three embryonic germ layers (ectoderm, mesoderm, and endoderm), and so, they give rise to all cells and tissues of the human body after a series of divisions and differentiations. The latter are present in the rest of the life of the embryo. They can contribute to all somatic and germline cell types when injected in the blastocyst and are also defined by their capacity to generate embryoid bodies *in vitro*, and teratomas *in vivo*. Accordingly, embryonic development and the subsequent stages of life can be understood as a continual loss of potencies.

Cardiac regeneration therapy through manipulation of ESC *in vitro* has been proved to be possible in animal models, with a growing body of evidence on isolation, differentiation, and application procedures [29]. The demonstration that ESC cells can differentiate into cardiomyocytes is the most important conclusion derived from several experimental studies. These cells can differentiate into cells with cardiomyocytic phenotype, with the same morphology, structural organization, and functional properties. They form sarcomeric structures and desmosomes, intercalated disks, and gap junctions, working as a functional syncytium with action potential propagation and spontaneous beating (i.e., electromechanical coupling with host myocardium). Thus, when delivered and transplanted into a zone of infarcted myocardium, these stem-cell-derived cardiomyocytes have been proved to engraft and improve left ventricular function [2, 30]. In CHF models, ESCs differentiate into new cardiomyocytes and other cell lineages necessary for the formation of new blood vessels (endothelial and smooth muscle cells), thus increasing the blood supply [31].

More recent studies have shown that pure cultures of cardiomyocytes can be created after genetic selection of ESC. These ESC-derived cardiomyocytes have expanding capacity and are suitable for transplantation [32]. Nevertheless, the use of ESC in clinical trials has several problems: ethical and legal issues, the requirement of immunosuppression in the case of an allogeneic origin, and the evidence of teratoma formation.

### 3.3. Adult Stem Cells

As well as in the embryo, stem cells are present in adult tissues of the postnatal organism as multipotent cells. Adult stem cells have been found in tissues with a rapid cell turnover, such as skin, liver, pancreas, and bone marrow, but also in skeletal muscle, intestinal mucosa, pancreas, heart, and central nervous system. In these tissues, they are committed to differentiate into mature functional cells and to integrate into each particular tissue and perform a specific function [25]. These cells stay in niches and differentiate into specialized cells of the same germ layer under certain circumstances and conditions. In these niches, adult stem cells require a specific microenvironment for their regulation, with special consideration for the extracellular matrix (ECM). Indeed, ECM has a critical role in stem cell self-renewal, proliferation, and growth. It is a storage depot for growth factors, hormones, and cytokines, and it uses integrins to communicate with cells. All these functions are lost after myocardial ischemia due to the release of matrix metalloproteinases (MMP) and cytokines from inflammatory and endogenous cells [24]. Thus, ECM and MMP also represent a possible target for therapeutic manipulation, as discussed later.

Adult stem cells are the most frequently used cells in clinical trials and in clinical regenerative research. They include the following cell types.

#### 3.3.1. Skeletal Myoblasts

Despite some authors do not consider skeletal myoblasts (SM) as true stem cells [7], they are indeed resident satellite stem cells [3]. They are committed solely to the myogenic lineage and have the ability for self-renewal and differentiation if muscle injury occurs. They are located between the basal lamina and the plasmalemma on the periphery of mature skeletal muscle fibers and remain in a quiescent stage until activated to divide in situations of wound or overburden. Therefore, they are a source of cells with contractile capacity able to completely regenerate and restore the cellular architecture in response to injury.

They can be easily obtained from muscular biopsies and expanded *in vitro*. Inasmuch as they have to be cultured and expanded for some weeks before transplantation, they cannot be used in the acute clinical setting [25].

Considerable attention has been paid to this sort of cells because of the following reasons: (1) they can be simplyobtained and amplified in an undifferentiated state with a great proliferation capacity; (2) they show good resistance to ischemic conditions; (3) they entail a source of autologous cells, not needing immunosuppressive treatments.

In accordance, transplantation of SM into injured myocardium has demonstrated increased survival and successful engraftment, differentiation into striated cells (myotubes and myocytes), and improvement of myocardial function by increasing infarcted wall resistance and stiffness. Nevertheless, there are some concerns [33]. Firstly, it is not clear whether these cells can survive for long periods of time in the host tissue. Secondly, SMs do not form new functional cardiac tissue: they cannot contract synchronously with the rest of the myocardial compartment because they do not form intercalated disks and electrical synapses with the host cardiomyocytes. This is so because when these cells differentiate into myotubes, they downregulate the expression of connexin-43 by a myoblast-induced upregulation of interleukin-1. Connexin-43 is important for normal formation of gap junctions, which are critical determinants of electrical stability. As a result, there is heterogeneity in the action potential duration between grafted cell clusters and host cardiomyocytes, and the incidence of arrhythmias is augmented. Other explanations for this increased risk of arrhythmias include the mode of cell delivery, the role of the underlying substrate (patients with previous myocardial infarction and depressed left ventricular function are supposed to have a high risk of arrhythmias), and the extent of myocardial tissue heterogeneity in the scar. Of note, the administration of SM after certain culture conditions (with autologous human serum) could avoid this risk [24].

#### 3.3.2. Bone-Marrow-Derived Cells

The bone marrow was the first used source of adult stem cells [9]. Nowadays, it can be considered the most investigated source and the one with the most extensive scientific evidence. The bone marrow is characterized by a complex architecture and specific geometric organization and harbors a heterogeneous population of cells: supporting mesenchymal cells (stromal cells), osteoblasts and osteocytes, vascular cells, adipocytes, and progenitor cells (stem cells). They all constitute an intricate system of cell-to-cell interaction and signaling.

In normal conditions, these progenitor cells are confined in the bone marrow, but they are also detected in the peripheral circulation. Indeed, hematopoietic growth factors like stem cell factor (SCF) and granulocyte colony stimulating factor (G-CSF) are known to facilitate the exit of these cells to the peripheral blood, as in situations of tissue damage [1].

Bone-marrow-derived stem cells can be collected by means of iliac crest aspiration, easily isolated on the basis of their adhesive properties, and expanded in culture. They can also be simply genetically modified, and finally administered to the patient with a number of diverse delivery strategies, either in the acute or in the chronic phase of ischemic cardiomyopathy. These cells are characterized by a great plasticity, showing even transdifferentiation into mature cells from different germinal layers (cardiomyocytes and vascular cells) [2, 29].

Furthermore, bone-marrow-derived stem cells have a very well-known secretor capacity. They are able to produce and release great quantities of angiogenic growth factors when cultured *in vitro*, such as basic fibroblast growth factor (bFGF), vascular endothelial growth factor (VEGF), and angiopoietins. It has been observed that bone-marrow-derived stem cells contribute to the formation of new endothelial cells in ischemic areas when injected in AMI animal models [7].

The most frequently used subpopulation of cells in cardiac stem cell therapy is the mononuclear fraction (BMMC). Precursors of both cardiomyocytes and endothelial cells exist within this cell fraction of the adult bone marrow, which includes MSCs, HSC, and EPCs. Other committed cell lineages are also included in the BMMC, such as natural killers, T and B lymphocytes. MSCs and EPCs represent a very small fraction of the total BMMC population (only 0.01% and 1–2%, resp.) and thus, culture techniques had to be developed in order to select and expand these specific types of cells [24].

EPCs and HSC are thought to result from a common precursor, the hemangioblast. Within the bone marrow, both immature cells express the surface markers CD34, CD133, and VEGF receptor 2 (VEGFR-2). When maturation goes toward the EPCs lineage, these cells lose CD133 but retain the two other markers, CD34 and VEGFR-2. Later, more differentiated EPCs express only endothelial lineage-specific markers like vascular endothelial (VE)-cadherin, platelet-endothelial cell adhesion molecule 1 (PECAM-1), von Willebrand factor (vWF), and E-selectine. On the other hand, when commitment to the hematopoietic lineage occurs, hemangioblast-derived cells no longer express CD133 nor VEGFR-2. HSC can be identified by the antigens CD34 and CD117 and the surface markers c-kit, Sca-1, and Thy-1 [34].

The transdifferentiation of HSC into cardiomyocytes and endothelial cells in the heart is widely accepted. Experiments with the administration of c-kit^+^/lin^−^ HSC after an AMI in animal models have demonstrated an evident increase in the number of cardiomyocytes in the infarcted area, but also improvements in myocardial perfusion, capillary density, and collateral vessel formation. As a result, these studies showed better cardiac function and survival benefits in treated animals [35].

EPCs have also been proved to be able to transdifferentiate into cardiomyocytes and smooth muscle cells *in vivo*. They can promote angiogenesis and vasculogenesis, increase capillary density, reduce cellular apoptosis and collagen deposit, and improve cardiac function. Nevertheless, these EPCs appear in low proportions in peripheral blood, and age and several pathologic conditions (i.e., risk factors) provoke a functional impairment of EPCs. On the other hand, some specific subpopulations of EPCs have interesting properties: CD14^+^/MAC-1^+^/CD11c^+^ EPCs have shown very high plasticity, and CD14^+^/CD34^+^ EPCs induce a paracrine response by releasing angiogenic growth factors [36].

MSCs constitute one of the most promising types of stem cells for cardiac repair [37]. They can be easily isolated and expanded, and they promote both neoangiogenesis and endogenous cardiac repair [9]. Moreover, they can be used in an autologous fashion and have an immunoprivileged profile: (1) they lack the expression of HLA class II and costimulatory molecules; (2) they prevent the T-cell response both directly by suppressing natural killer, CD4^+^ and CD8^+^ T-cell function, and indirectly through modulation of dendritic cells; (3) they can induce a suppressive local microenvironment through the production of prostaglandins and interleukins. They have been isolated from the bone marrow stroma, but can also be found around blood vessels, in skeletal muscle, skin, and adipose tissue. MSCs are characterized by an immunophenotype positive for adhesion proteins like CD29, CD44, CD71, CD90, CD105, CD106, CD117, CD120a, CD124, SH2, SH3, and SH4, and thus, they are isolated by means of their ability to adhere to culture plates [38]. Interestingly, MSCs exhibit a multipotent differentiation capacity: purified human MSCs have been shown to migrate and differentiate into a cardiomyocytic phenotype and into endothelial cells in both healthy and infarcted myocardium [39]. In the former, MSCs express cardiac surface markers, and in the latter they improve wall motion and prevent the adverse remodeling process. Apart from the important differentiation capacity of these cells, potent paracrine effects by MSCs have also been demonstrated [40].

#### 3.3.3. Adipose-Derived Stem Cells

Recently, a population of stem cells has been identified in the stroma of the adipose tissue: multipotent adipose-derived stem cells (ADSCs). The phenotype of these cells is similar to that of MSCs from the bone marrow; they express adhesion molecules in their surface and possess high potentiality. They are CD34^+^, CD105^+^, CD45^−^, and CD31^−^ cells that also express mesenchymal markers such as CD73 and CD90 [41]. They are capable of differentiating into myogenic, neural, and cardiomyocytic lineages, in this last case showing even spontaneous beating [2]. Vascular endothelial growth factor (VEGF) seems to contribute to the differentiation of this kind of stem cells into cardiomyocytes.

The key point of the use of adipose tissue as a novel promising alternative source of stem cells for cardiovascular repair is that this tissue can be easily harvested by liposuction procedures and is a rich source for multipotent cells, including hematopoietic, endothelial, and mesenchymal progenitor cells, apart from ADSCs [25]. Moreover, experimental data suggest that these cells augment neovascularization and improve cardiac function after an AMI in a similar magnitude to that demonstrated with bone-marrow-derived cells.

#### 3.3.4. Resident Cardiac Stem Cells

Another sort of stem cells has been recently identified in both normal and pathological adult hearts, the so-called resident cardiac stem cells (CSCs) [1–3]. Recent elegant studies have confirmed that cardiomyocytes are renewed in the adult heart at a rate of 1% per year at the age of 25 and of 0.45% at the age of 75. Thus, at the age of 50, 55% of the cardiomyocytes remain from the time around birth and 45% have been generated later from the pool of CSC [42]. Having said that and in spite of their presence in the adult heart as the responsible for the “natural” myocardial repair, CSC number is not enough to achieve a spontaneous total recovery after myocardial injury.Resident CSC are thought to occupy specific niches in the atria, in the ventricular base, and in the apex, from where they can be obtained by cardiac biopsy. In pathological conditions, they have also been observed in the border zones of myocardial infarctions [25].

Related to CSC, another interesting subpopulation of progenitor cells has been demonstrated in the epicardium and named “epicardium-derived” progenitor cells (EPDCs). These cells are able to stimulate the growth of various endothelial cell lines and to secrete proangiogenic factors [43].

CSCs express surface markers such as the transmembrane tyrosine kinase receptor c-kit and CD117 (which best characterize these progenitor cell-type), Sca-1, and Abcg2. Clonal c-kit^+^ population can regenerate all lineages of the heart, and their number increases after myocardial injury, as do Sca-1^+^/CD31^−^ cells. They can contribute to myocardial regeneration and repair as shown in animal models of AMI, accordingly to their capability to differentiate into cardiomyocytes and vascular, smooth muscle, and endothelial cells. Also, in the adult human heart, CSC can differentiate into cardiovascular lineages and consequently improve cardiac function when transplanted into ischemic hearts [44, 45].

It has been suggested that ischemic myocardial injury induces the activation of CSC, through signaling mediated by cytokines and growth factors, which trigger the activation and proliferation of these endogenous cardiac precursors. Furthermore, exogenous-administered stem cells could also contribute to the activation and restoration of the CSC niches through paracrine mechanisms, facilitating and maximizing the ability of the heart to repair itself [29]. Also and similarly to what has been described for bone-marrow-derived EPCs, cardiovascular risk factors, patient characteristics, cardiac function, and pharmacologic therapy appear to modify and modulate myocardial homeostasis and CSC therapeutic capacity. For example, in murine models, a positive and significant correlation between the number of CSC and betablocker treatment and between c-kit expression and pulmonary hypertension has been observed. C-kit^+^ cell amplification potential directly correlated to pulmonary hypertension and statins intake, whilst it inversely correlated to smoking, atrial fibrillation and previous myocardial infarction [46].

#### 3.3.5. Umbilical Cord Blood Stem Cells

The umbilical cord blood contains a large proportion of hematopoietic and mesenchymal precursor cells and in higher numbers than in peripheral blood or in the bone marrow [1]. Also known as “somatic nonrestricted stem cells”, these cells are negative for c-kit, CD34, and CD45, are fibroblast-like in appearance, and adhere to culture dishes. In animal models of AMI, they differentiate into cardiomyocytes, improve ventricular perfusion and contractility, and reduce the infarct size [47]. Nevertheless, cord blood stem cells have not been used in clinical trials so far because of ethical and legal issues.

### 3.4. Induced or “Embryonic-Like” Stem Cells

The generation of induced pluripotent stem cells (iPS) lines derived from adult human cells was first reported in 2007 [48, 49], when nuclear reprogramming to convert somatic cells into stem cells was proved to be feasible. Thus, differentiated mature somatic human cells can effectively be reprogrammed into a pluripotent state by transduction of four defined transcription factors (human stemness factors OCT3/4, SOX2, KLF4, and c-MYC) [50]. The resulting iPS clones, with inherent cardiogenic potential, were proved to have the same morphological characteristics, surface markers, proliferative capacity, and potentiality as those known in ESC. iPS can differentiate into any type of cell from any of the three embryonic germ layers [1]. Until 2009, only 3 disease models had been treated with this strategy (sickle cell anemia, Parkinson's disease, and hemophilia A). Since then, several studies have demonstrated the efficacy of these cells in the setting of myocardial infarction, by contributing to multilineage regeneration and by reducing scar size, as well as improving cardiac remodeling at global, regional, and electric levels.

On the other side, it has also been observed that cardiomyocytes and MSCs can be generated from iPS, via a promesoderm differentiation strategy. MSCs generated from iPS are capable of lineage-specific differentiation, showing a robust growth potential and a marked proangiogenic capacity. More importantly, they appear to be free from cytogenetic abnormalities and tumorigenesis risk [51].

iPS cells were first generated from mouse and human fibroblasts. Going further in this field, generation of iPS cells from cardiac-ventricular-specific cell types (such as H9c2 cells) has been achieved using the same transcription factors. These iPS cells were able to differentiate into beating cardiomyocytes and positively stained for cardiac specific proteins. Following transplantation in the infarcted myocardium, there were newly differentiated cardiomyocytes and formation of gap junction proteins at 2 weeks after myocardial infarction, proving that newly formed cardiomyocytes were integrated into the native myocardium. Furthermore, transplanted iPS cells inhibited apoptosis and fibrosis, improved cardiac function, and showed a differentiation potency *in vitro* and *in vivo* which was comparable to ESC [52].

However, two safety concerns have been pointed out with the use of iPS: firstly, the risk of mutations after viral transfection: secondly, the risk of teratoma formation. In consequence, it seems realistic to think that further investigations are necessary before their application in human beings.

## 4. Cell Lineages Needed for Cardiac Repair

As we have seen, there is strong evidence that the heart muscle has the ability to regenerate itself through the activation of resident CSC or through the mobilization and recruitment of stem cells from other tissues. Nevertheless, this regenerative capacity is overwhelmed by tissue loss after cardiac injury and cannot compensate that loss.

Stem cell therapy has accumulated growing evidence in different pathophysiological conditions in small and large animal models, but human research has been almost limited to ischemic cardiomyopathy.

Natural history of CAD can be divided into AMI and chronic ischemic heart disease. Potential benefit of stem cell therapy after myocardial infarction seems to be related to angiogenesis, decreased apoptosis of native cardiomyocytes, and enhanced collagen formation, which altogether may limit infarct expansion and preserve myocardium [2]. Accordingly, the negative left ventricular remodeling process after myocardial infarction may diminish or reverse, leading to a stabilization of ventricular dimensions and to a normal ventricular function [53].

In the chronic phase of myocardial ischemia, stem cell therapy has been especially investigated in two fields: pump failure due to ischemic ventricular dysfunction [33] and refractory angina.

When the aim is to restore contractile function (end-stage ischemic CHF or early postinfarction), delivering cells with contractile potential may be of high priority. In such conditions, those cellular lines capable of giving rise to myogenic cells (i.e., SM, cardiomyocytes, or any progenitor cell driven down a muscle lineage) appear to be the first choice. Formation of new myocardial mass has only been strictly established for ESC andhas been achieved in few trials and in small percentages with adult stem cells.

When chronic ischemia prevails, a more reasonable approach would be the use of cells with angiogenic potential. In this case, BMMC, EPCs, vascular progenitor cells or blood-derived multipotent adult progenitor cells, and MSCs may be better choices than myogenic precursors.

### 4.1. Stem Cell Therapy after Acute Myocardial Infarction

In the setting of an ischemic event, there is an immediate and massive infiltration of circulating leukocytes into damaged tissue. Endogenous cells suffering ischemia release cytokines and chemokines (interleukin 1 [IL-1], interleukin 6 [IL-6], interleukin 8 [IL-8], and tumor necrosis factor [TNF]) and upregulate cell adhesion molecules in endothelial cells (E-selectin). Also, the infiltration of myofibroblasts contributes to collagen and ECM's protein deposit, promoting scar formation.

Stem cell therapy in this scenario aims at two main objectives: firstly, recruiting and facilitating the homing of endogenous or circulating stem cells with locally injected growth factors, cytokines, and other molecules: secondly, replacing dead cells by local-transplanted stem cells, capable of producing new cells.

Several trials have evaluated stem cell therapy after AMI, some with positive results and some others with neutral ones. Most of the studies used BMMC, delivered in an intracoronary fashion after restoration of the infarct-related artery patency. Four main RCTs have been published with positive findings (REPAIR-AMI [54], BOOST [55], FINCELL [56], and REGENT [57]), showing an increase in left ventricular ejection fraction (LVEF). On the other hand, three RCTs resulted in neutral findings, with no apparent benefits obtained from this therapy (ASTAMI [58], HEBE [59], and the study by Janssens et al. [60]).Importantly, no safety concerns after BMMC intracoronary infusion have emerged: none of the trials reported an increased incidence of malignant arrhythmias.

Two trials have used MSCs after AMI. The study by Chen et al. demonstrated an improvement in LVEF and perfusion with intracoronary infusion of these cells [61], but these results have not been duplicated. Hare et al. intravenously administered allogeneic MSCs after an AMI with no higher rates of adverse events and some benefits in terms of LVEF [62].

New types of cells are also being explored, like ADSCs. No evidence is available to date, but the first in-man clinical trial with intracoronary administration of freshly isolated ADSCs after AMI (the APOLLO trial) has been recently completed.

Another approach for stem cell therapy after AMI is cell mobilization from the bone marrow with the administration of G-CSF. Several clinical trials have been published, but results have been less encouraging. Only three trials have reported positive results with significant improvements in LVEF (FIRSTLINE-AMI [63], RIGENERA [64], and the study by Takano et al. [65]). The rest of the trials showed negative findings.

Finally, the MAGIC trials used a combination of G-CSF and intracoronary injection of peripheral blood progenitor cells. In the first trial, no differences in LVEF were noted, and an increase in in-stent restenosis rates was observed (G-CSF was administrated before bare-metal stent implantation) [66]. Then the investigators changed the design and used drug-eluting stents. In the MAGIC 3-DES trial, positive results in terms of LVEF were found after mobilization and intracoronary injection of isolated cells [67].

### 4.2. Stem Cell Therapy for Chronic Ischemic Heart Disease

#### 4.2.1. Ischemic Heart Failure

SM and BMMC have been used in CHF patients. The MAGIC trial [68], with transepicardial injection of SM during coronary artery bypass graft (CABG) surgery, reported no changes in global or regional contractility. However, a reduction in left ventricular end-diastolic and end-systolic volumes was observed in the high-dose group. Moreover, a trend towards a higher incidence of ventricular arrhythmias was noted. Dib et al. reported an improvement in LVEF and viability after SM transendocardial injection [69], in contradiction with the SEISMIC trial, which showed no benefit of the same procedure.

In the TOPCARE-CHD trial, BMMC intracoronary delivery into the coronary artery supplying the most dyskinetic left ventricular area showed improvements of the LVEF [70]. Although it lacks a randomized design, the STAR-heart study represents the largest trial with BMMC in patients with severe left ventricular dysfunction [71]. Intracoronary transplantation improved haemodynamics at rest including LVEF, exercise capacity, left ventricular contractility and geometry, and mortality at 5-year followup.

Interestingly, CSCs have also been investigated in this scenario. In the CADUCEUS trial, patients treated with intracoronary infusion of CSC 1.5–3 months after AMI showed reductions in scar mass, increases in viable heart mass and regional contractility, and regional systolic wall thickening with no changes in end-diastolic volume, end-systolic volume, and LVEF [72]. In the SCIPIO trial, the injection of CSC while coronary artery bypass grafting (CABG) resulted in improvements of both LVEF and infarct size [73].

#### 4.2.2. Chronic Myocardial Ischemia

Patients with advanced CAD and no further options of revascularization (“no-option” patients) have also been studied in stem cell therapy trials. Three RCTs have been completed using the transendocardial route, with BMMC or blood-derived progenitor cells. Angina frequency and exercise time were improved, but no clear effects on myocardial perfusion were observed. In the PROTECT-CAD trial, BMMC injections improved NYHA functional class, exercise time, LVEF, wall thickening, and stress-induced perfusion defects [74]. Van Ramshorst et al. reported better LVEF, myocardial perfusion, angina functional class, exercise capacity, and quality of life after BMMC administration [75].

ADSCs have also been studied in this type of patients. The PRECISE trial is a RCTs in which 27 no-option patients with left ventricular dysfunction were randomized to receive freshly isolated ADSCs or placebo. The cells were delivered via transendocardial injections after left ventricular electromechanical mapping, and results are still waiting for publication.

## 5. Strategies for Cell Potency Enhancement

Although evidence about stem cell therapy benefits has grown exponentially during the last years, uncertainty persists regarding the way progenitor cells act. Stem cells could exert their benefit by any or a combination of three main general mechanisms: (1) differentiation of the administered cells into all of the cellular constituents of the heart (i.e., cardiomyogenesis and vasculogenesis processes), or, less probably, fusion of the administered cells with those, (2) release of factors capable of paracrine signaling from the administered cells, and (3) stimulation of endogenous repair by injected cells, through stem cell cardiac niches activation [6, 24]. None of these three mechanisms is still completely understood, and consequently a more profound knowledge on these mechanisms of action is warranted in order to improve clinical results [5].

On the other hand, cell engraftment and survival after transplantation are essential for cardiac repair. However, different studies indicate that both phenomenas are very limited after cell transplantation into damaged myocardium, because the ischemic environment in which cells are deployed makes them disappear within a few days. In this regard, three fields of research are well known to be improvable: (1) adequate *in vitro* stem cell expansion prior to transplantation, (2) optimal timing, and (3) dose and delivery methods of cell transfer. In the next section we will summarize the current lines of research related to cell multipotency.

### 5.1. Strategies to Increase Cell Potency

Of the three aforementioned mechanisms of action of stem cells, differentiation of the administered cells into cardiac tissue seems the less plausible one because exogenous cells survive in very low numbers and during very limited periods of time in the ischemic myocardium. This has been the starting point for the development of different strategies aimed at improving the differentiation capacity of stem cells while they remain alive and functional in the myocardium [24], among them are the following.

1. Pretreatment of the patients with drugs to stimulate cell potency: statins, rosiglitazone, and the nitric oxide synthase enhancer AVE9488 can improve the migratory, invasive, and neovascularization capacity of EPCs.
2. Strategies to prolong cell survival: among them, the use of a combination of growth factors to stimulate the expression of cardiomyocyte genes in MSCs, the use of heat shock to increase the resistance of cells to external stressors, and the pretreatment of ESC-derived cardiomyocytes with heat shock and a cocktail of survival factors are being explored.
3. Genetic modification of the cells prior to administration: overexpression of antiapoptotic genes (Bcl-2, Akt or hemoxigenase-1) or genetic manipulation to maintain cell functionality (i.e., capacity to secrete paracrine mediators, to connect with host myocardium, or to differentiate into specialized cardiac cell types) can be achieved through genetic cell engineering.

### 5.2. Strategies to Increase Cell Retention and Survival

Low-perfusion conditions that define ischemic tissues generate poor conditions for the transplanted cells, which are delivered in a hostile environment characterized by inflammation, oxidant stress, and cytotoxic cytokines. The following innovations, although not fully related to differentiation potency of the cells, are being developed in order to increase cell engraftment and survival rates [76–78].

1. Preconditioning of the myocardium to retain a higher number of cells: low-energy shock waves, ultrasound-mediated destruction of microbubbles in the coronary circulation, and extracorporeal shock wave treatment have proved to increase retention of EPCs, BMMC, and MSCs.
2. Activation or increase of chemotactic factors to attract cells to the damaged area: high mobility group box-1 (HMGB-1), SDF-1 or its receptor CXCR4, *β*2 integrin and endothelial nitric oxide synthase can be activated to increase the rate of homing of different types of stem cells (i.e., progenitor blood cells and EPCs). Conversely, it is now well known that reduced expression of CXCR4 in the setting of AMI entails a loss of functional benefit of MSCs [79].
3. Combined injection of cells and biomaterials: BMMC encapsulation within scaffolds (epicardial patches) or peptide nanofibers represents another strategy that is being investigated. An appropriate scaffold for cardiac repair should have specific properties: noncytotoxicity, low-inflammatory and immunological response, and biodegradability, to facilitate the integration of the transplanted cells. Size and texture must be also taken into account, as they determine cell retention in the tissue. Going further, the process of tissue decellularization with detergents has demonstrated that it is possible to obtain “acellular” extracellular matrixes—with the entire cardiac architecture—where CSC or EPCs can be reseeded.
4. Formation of synthetic or cellular spheres: pharmacologically active microcarriers (PAMs) are biodegradable and noncytotoxic poly (lactide-co-glycolide) microspheres covered with ECM molecules used to create a three-dimensional microenvironment in which cells can be transplanted *in vitro* and *in vivo*, enhancing their engraftment and integration [80]. On the other hand, “natural” cardiospheres can be obtained with CSC. This technique involves a special culture strategy by which CSCs form primary and secondary cardiospheres [81], eliminating differentiated cells and selecting a core of c-kit^+^ cells surrounded by differentiated cardiomyocytes at the surface [41]. These cardiospheres have revealed greater cell engraftment and survival than CSC alone and have been associated with improvements in ventricular function and remodeling after AMI.
5. Genetic modification of the host tissue: myocardial fibrosis is known to act as a barrier that impairs penetration of progenitor cells mobilized in response to cardiac damage. In murine models, overexpression of adenylyl-cyclase 6 (AC6) in cardiac cells reduces collagen density in scar tissue, facilitating iPS engraftment and increasing angiomyogenesis and cardiac function [80].

## 6. Conclusions

Differentiation capacity is the most genuine characteristic of stem cells. During the past two decades, hundreds of studies have demonstrated that processes of differentiation and transdifferentiation are of great magnitude in small and large animal models of ischemic cardiomyopathy and produce significant improvements in cardiac perfusion and function.

However, these processes have been observed in humans in very low proportions, and paracrine mechanisms have been invoked as the main mediators of stem cell benefits. More experimental and preclinical studies are needed to shed light on the molecular and cellular basis of stem cell differentiation, especially after their administration in humans. The complete knowledge of this fascinating and complex network of intra- and intercellular mechanisms and interactions will lead us to another era in cardiovascular regenerative medicine, in which the best cell types will be modified to augment their reparative potential and to produce optimal benefits for damaged human hearts.
